# Stable or fluctuating temperatures in winter: which is worse for your lungs?

**DOI:** 10.1136/thoraxjnl-2018-211898

**Published:** 2018-06-22

**Authors:** Kin Bong Hubert Lam

The ratification of the Paris Agreement in 2015^1^ signifies a worldwide consensus on climate change has been reached. The Intergovernmental Panel on Climate Change has warned as global warming intensifies, extreme weather events (such as heat waves, droughts, floods) will become more frequent and more severe.^2^ Indeed in the UK, within the first few months of 2018, there has already been a cold wave between late February and early March (with maximum temperature 8°C–12°C below average for the time of year)^3^ and an unusually warm week in April (which saw a rise of almost 20°C from 6°C–7°C in the previous week).^4^ That heat waves or cold spells could lead to excess deaths has been documented in previous studies^5–7^ and is probably unquestionable. However, even in the absence of extreme temperature episodes, exposure to so-called non-optimum ambient temperatures has shown to be associated with elevated risks of morbidity and mortality.^8–10^ A recent study of over 74 million deaths from 13 countries has found most of the temperature-related short-term (days to weeks after the exposure) mortality displacement could in fact be attributed to moderate hot and cold temperatures, rather than extreme heat (temperature higher than 97.5th percentile) or cold (lower than 2.5th percentile).^9^

Many of these previous studies have used mean daily temperature as the exposure metric. The fact that health impact has been observed in both colder and hotter temperatures suggests there is adaptation to usual temperatures and unexpected fluctuations in temperature may be more relevant. Temperature variability, as it is known, can be measured in different ways but mostly focusing on intraday (diurnal temperature range) or interday (change between neighbouring days) variability.^11–13^ Effects of longer term temperature variability, usually defined as SD of the daily mean temperatures in summer and/or winter, have also been examined in the US population with significant associations with mortality observed.^14–16^

Sun and colleagues^17^ report, for the first time, the association between seasonal temperature variability and the first occurrence of respiratory disease hospitalisation, based on the data from a large prospective population-based cohort of 61 446 older Chinese individuals in Hong Kong with 10–13 years of follow-up. They were able to assign individual ambient temperature exposure according to their residential address, by interpolating measurements from the territory-wide network of 22 weather stations using ordinary kriging (a commonly used geospatial algorithm). Despite the relatively small SD of temperature during summertime (June–August; mean: 1.4°C) and wintertime (December–February; 3.2°C), after adjusting for a range of confounders both at individual and ecological levels, they found a significant association between wintertime temperature variability and emergency respiratory disease admissions (HR 1.20, 95% CI 1.08 to 1.32 for each 1°C increase in temperature variability), largely contributed by chronic obstructive pulmonary disease (COPD) (HR 1.41, 95% CI 1.15 to 1.71) and pneumonia (1.15, 1.01 to 1.31). Such associations did not appear to be influenced by reverse causality and confounding by fine particulate air pollution, and were slightly stronger in women and those who had less personal monthly expenditure. Unlike the previous American mortality studies,^15 16^ there was no association with summertime temperature variability. However, this may not refute their findings as the authors explain people in Hong Kong may be accustomed to the hot and humid subtropical summers (and perhaps to some extent eased by air-conditioning) and be more sensitive to cold weather.^17^

Sun and colleagues^17^ acknowledge that their study, like others examining the health impact of temperature, has major limitations in exposure assessment. Thermoregulation of the human being involves multiple complex physiological mechanisms.^18^ While ambient air temperature is a key determinant, its interplay with behavioural, lifestyle-related and socioeconomic factors is equally important. For example, apparent temperature, which is the temperature perceived by the body, takes into account wind speed and relative humidity. Thus, the physical environment (eg, sheltered or exposed) where an individual is may affect how he/she reacts (eg, switching on air-conditioning or heater, adjusting physical activity level), which may ultimately alter his/her personal exposure level. In elderly populations such as the one studied by Sun and colleagues,^17^ it is not uncommon that they tend to be housebound (or institutionalised) due to frailty and multimorbidities (in this study, 53% took regular medication at baseline). Urban heat island effect, characterised by higher temperatures in cities compared with their neighbouring rural areas, is particularly relevant to Hong Kong due to its extremely high density of high rises in a number of small pockets of built-up areas.^19^ Local-scale or microclimate urban heat islands can develop within neighbourhoods and street canyons as a result of human of activities and topography. Vertical living in Hong Kong also introduces another layer of complexity—elevation. Nevertheless, many of the weather monitoring stations, from which ambient air temperature data in the study by Sun *et al*^17^ were derived, are housed in parks and open spaces,^20^ such that underestimation of urban temperatures, especially within residential high rise communities, is likely.^21^ Exposure misclassification will remain to be a major challenge unless future studies are able to take advantage of technology (eg, temperature sensing module to be used with smart phone, smart thermostat) to collect real-time personal temperature data in the big data era.

Another question remains: what does seasonal temperature variability actually mean? In the current definition, it is simply the SD of all mean daily temperatures within the specified season. It condenses direction (lower to higher temperature or vice versa), rate (how long it takes to reach higher/lower temperature and the cumulative gain/loss) and frequency (and stability) of fluctuations (say low-high-low-high or low-low-high-high) all into one single index. So, which is more relevant to lung health, stable (sustained cold/hot temperature) or fluctuating temperatures? The study by Sun *et al*^17^ does not seem to give the answer, although the estimated effect of temperature variability on hospitalisation is independent of yearly mean temperature.

Readers have to be careful in interpreting the study. The endpoint chosen is the first occurrence of emergency admission after enrolment, rather than *first ever* pneumonia or COPD hospitalisation as the word ‘incident’ might often be interpreted. In this study as many as 62% (1896 (from table 3)/3075 (from table 2), assuming self-report was complete and asthma prevalence was negligible) of the COPD cases were diagnosed after baseline. As COPD is often insidious and may take many years before it is diagnosed, especially in this cohort where female smoking is less common (88% never smokers),^22^ it is possible that individuals with newly diagnosed COPD were not aware of their condition and had not received proper management and would be hit harder by temperature variability (modest attenuation of HR estimate in the sensitivity analysis when excluding self-reported COPD/asthma). This perhaps further highlights the importance of self-adjustment and adaptation, which have been mostly ignored (or unable to be accounted for) in the analyses so far.

For now, while we are still contemplating whether wintertime temperature variability is a proxy indicator of (or enabling factor for) respiratory infection-related hospitalisations, we should start to consider what advice and support should be given to the vulnerable populations and whether our current built environment may withstand the test of climate change.
